# Europe’s lost forests: a pollen-based synthesis for the last 11,000 years

**DOI:** 10.1038/s41598-017-18646-7

**Published:** 2018-01-15

**Authors:** N. Roberts, R. M. Fyfe, J. Woodbridge, M.-J. Gaillard, B. A. S. Davis, J. O. Kaplan, L. Marquer, F. Mazier, A. B. Nielsen, S. Sugita, A.-K. Trondman, M. Leydet

**Affiliations:** 1 School of Geography, Earth and Environmental Sciences, Plymouth University, Drake Circus, Plymouth, UK; 2 Department of Biology and Environmental Science, Linnaeus University, Barlastgatan 11, Kalmar, Sweden; 3 Max Planck Institute for the Science of Human History, Jena, Germany; 4 Department of Physical Geography and Ecosystem Science, Lund University, Sölvegatan, Lund, Sweden; 5 Bureau Research House of Jean Jaurès University, 5, Allées A. Machado, Toulouse, C229 France; 6 Institute of Ecology, Tallinn University, Narva mnt 25, Tallinn, Estonia; 7 Institut Méditerranéen de Biodiversité et d’Ecologie marine et continentale, Aix-Marseille Université, Marseille, France; 8 ARVE Research SARL, Pully, Switzerland; 9 Institute of Earth Surface Dynamics, University of Lausanne, Geopolis Building, Lausanne, Switzerland

## Abstract

8000 years ago, prior to Neolithic agriculture, Europe was mostly a wooded continent. Since then, its forest cover has been progressively fragmented, so that today it covers less than half of Europe’s land area, in many cases having been cleared to make way for fields and pasture-land. Establishing the origin of Europe’s current, more open land-cover mosaic requires a long-term perspective, for which pollen analysis offers a key tool. In this study we utilise and compare three numerical approaches to transforming pollen data into past forest cover, drawing on >1000 ^14^C-dated site records. All reconstructions highlight the different histories of the mixed temperate and the northern boreal forests, with the former declining progressively since ~6000 years ago, linked to forest clearance for agriculture in later prehistory (especially in northwest Europe) and early historic times (e.g. in north central Europe). In contrast, extensive human impact on the needle-leaf forests of northern Europe only becomes detectable in the last two millennia and has left a larger area of forest in place. Forest loss has been a dominant feature of Europe’s landscape ecology in the second half of the current interglacial, with consequences for carbon cycling, ecosystem functioning and biodiversity.

## Introduction

Along with an intrinsic interest in Europe’s natural and cultural heritage, there is a need by climatologists, archaeologists, geomorphologists, conservation ecologists and others for the reconstruction of long-term, large-scale changes in forest cover, especially those associated with human activities^1^. For example, pollen-based reconstructions potentially provide a critical empirical test of the hypothesis that human land-cover change already started to alter greenhouse gas emissions significantly, and thereby global climate, thousands of years ago^2^. Although many previous studies have investigated how pollen can be used to infer past forest cover, most have been site- or region-specific^3–5^. Spatially-extensive (e.g. Europe-wide) syntheses have focused instead on what pollen can tell us about changes in vegetation species composition, biome distribution or climate^6,7^, rather than explicitly incorporating human-induced changes in forest cover. Recently, a number of different numerical approaches have been developed to reconstruct large-scale past land cover from pollen data. They include the REVEALS model^8^ linked to the LandClim project^9–11^, the Pseudobiomization Method (or PBM^12–15^ and approaches based on Plant Functional Types (PFTs^16^).

**Figure 1:**
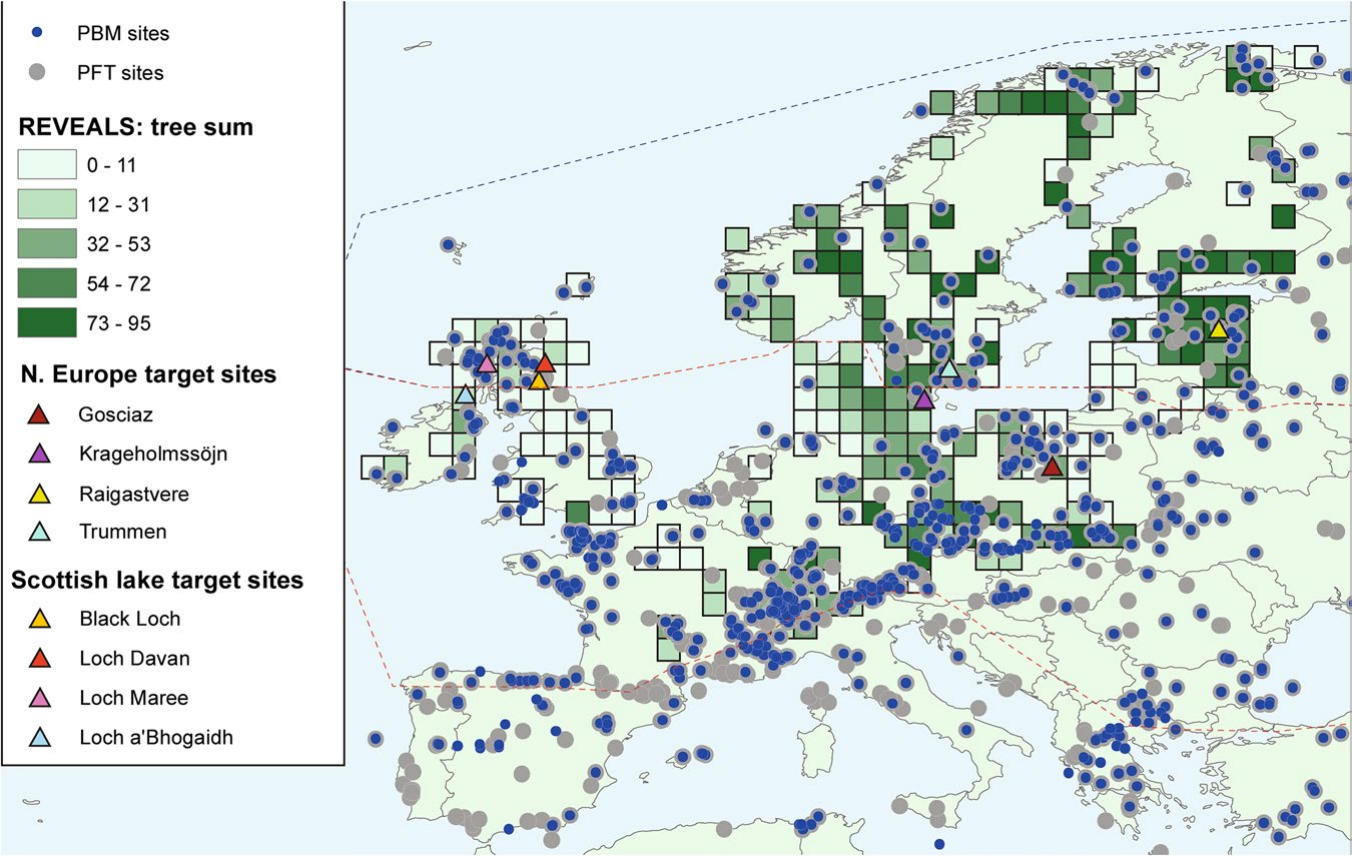
Map of Europe showing sub-divisions used, and grid cells map for mid-latitude (mixed temperate forest zone) and northern (boreal needle-leaf forest zone) Europe. [map generated using ArcMap (version: 10.4) URL: http://desktop.arcgis.com/en/arcmap/].

The REVEALS (Regional Estimates of VEgetation Abundance from Large Sites) approach of Sugita^8^ is a generalized version of the r-value model of Davis^17^, and accounts for taxon-specific differences in both relative pollen productivity and pollen dispersal properties. REVEALS provides a quantitative estimate of the regional abundance of plant taxa at a spatial scale of ca. 100 × 100 km from pollen sites that have a large recruitment area. It has been evaluated against modern vegetation surveys for a number of study areas (e.g. southern Sweden^18^ and North America^3^) and has resulted in various regional-scale syntheses of Holocene land cover during the Holocene^4,19–21^. Whilst there is a high degree of confidence in the results of the REVEALS approach, it is a complex method that relies on the availability and reliability of a key input parameter, the relative pollen productivity (RPP) estimate of the various taxa under study^22^. Because of the limited number and coverage of RPP studies to date, published results from REVEALS analysis are presently available for selected areas of mid-latitude and northern Europe (Fig. 1). The Europe-wide synthesis of 1 × 1° grid-based woodland cover of Trondman *et al*.^10^ covers five time windows, namely 6200–5700, 3200–2700, 700–350, 350–100 and 100 to −65 yr BP (calendar years Before Present, defined as 1950AD), although full Holocene reconstructions at a time resolution of 150–500 years have been generated from Trondman *et al*.’s wider study region^4,19,21^.

Both PBM and PFT approaches are intermediate in complexity, and do not take full account of taxon-specific differences in pollen productivity and dispersal. The PBM transforms pollen proportions from each site into a number of land cover classes (LCCs^12,13^). Fyfe *et al*.^12^ applied the PBM to data taken from the European Pollen Database^23^ at 200-year time steps, producing three closed forest type LCCs, and a fourth semi-open forest LCC, i.e. a mix of open and forest vegetation. Two different PBM values are provided here, firstly, the closed forest sum (= PBM score, abbreviated as PBM_sc_); secondly, percentage of samples assigned to forested Land Cover Classes (= LCC assigned, abbreviated as PBM_lcc_)(see methods). The PFT approach groups pollen taxa into plant functional types and changes in landscape openness are assessed by calculating the arboreal pollen PFT percentage. Davis *et al*.^16^ applied this method to data from the EPD in 500-cal. year time steps. The PFT and PBM approaches only provide a semi-quantitative estimate of the past areal extent of forested land. On the other hand, both PBM and PFT approaches offer continuous reconstructions of Holocene vegetation change at multi-centennial time resolution, and have good spatial coverage over most of Europe.

Given that each approach has strengths and weaknesses, there is value in assessing how far their results agree or diverge. Such an assessment is needed, for example, if pollen-based reconstructions are to be used to evaluate anthropogenic land-cover change scenarios (ALCCs) such as KK10^24^ or HYDE^25^, particularly as these computer model simulations do not agree on the severity or scale of human-induced land-cover change. Congruence – or lack of it - between pollen-inferred and computer-simulated forest cover will be more meaningful if the former are demonstrably robust, and this may be achieved via inter-comparison of different pollen-based approaches. In this study we have undertaken a systematic comparison of past forest cover using REVEALS, PBM and PFT approaches in order to assess when and where Europe’s primaeval wildwood was transformed into today’s semi-natural landscapes and habitats. We divided the study region into mid-latitude and northern Europe. The boundary between the two sub-regions was drawn between the temperate and hemi-boreal vegetation zones as defined by Ahti *et al*.^26^ for northern Europe (except for Scotland where the Lowland-Highland divide was used). Because of limited pollen data coverage, we do not attempt to make forest cover reconstructions for the area east of 32° E. This region includes the major share of land that was never occupied by woodland because of low precipitation. Similarly, because of the lack of REVEALS reconstructions for Mediterranean Europe, no comparison has been attempted here for this southern region of Europe. In addition, we draw on further data sets for modern forest cover, namely the surface pollen data set of Davis *et al*.^27^ and two remote sensing datasets, a 100 m resolution CORINE land-cover map^28^ and 25 m resolution Forest MAP 2006^29^.

## Results

**Figure 2:**
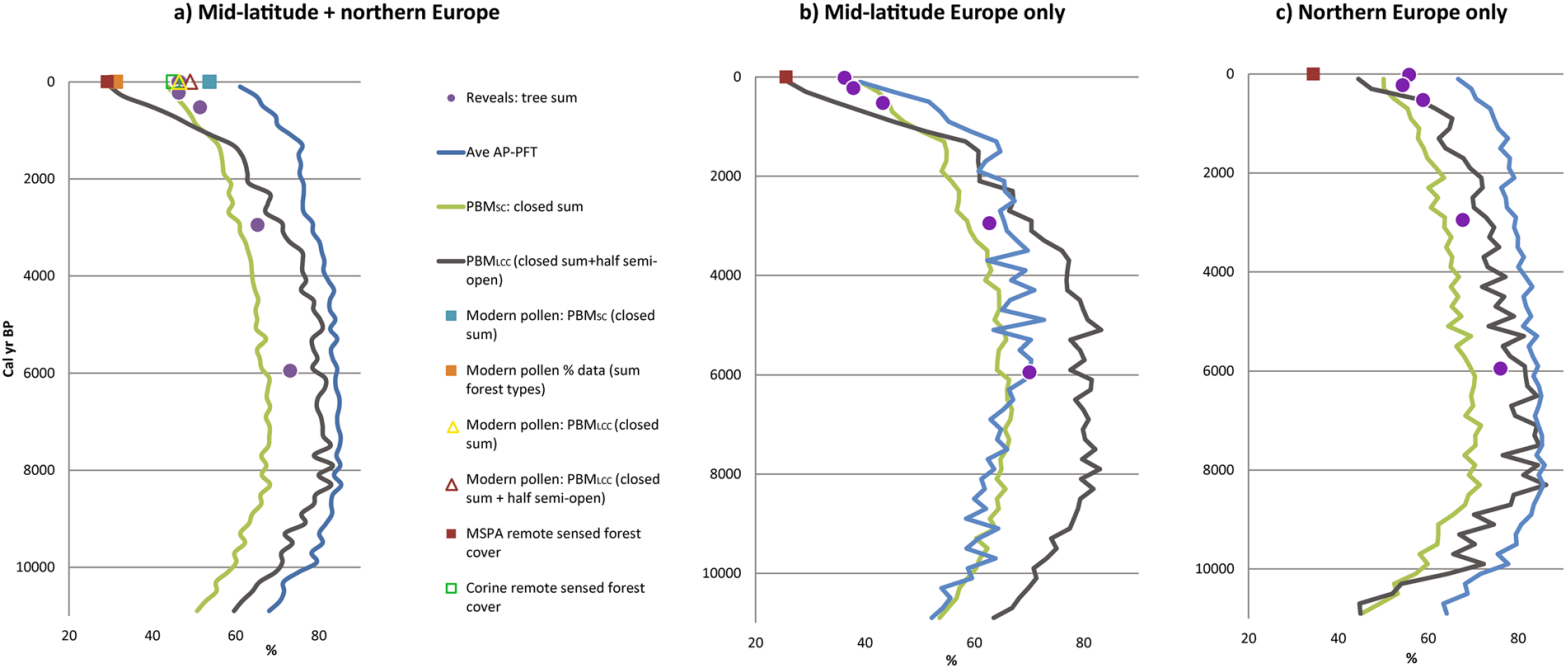
Pollen-inferred forest cover for mid-latitude and northern Europe during the last 11,000 years (REVEALS grid cells only).

As shown in Fig. 1, there is good spatial coverage for the three pollen-derived data sets north of the Alps from Ireland to the Baltic. The time-continuous reconstructions for these mid-latitude and northern European grid cells based on the PBM and PFT approaches are shown in Fig. 2. They show a common trend of increasing forest cover from >11,000 to ~8000 yr BP, a forest maximum between 8000 and 6000 yr BP, and a gradual decrease in forest cover since the mid-Holocene. The curves based on PFT and PBM_sc_ show parallel trends, although the PFT method has forest sum values consistently 15–20% higher than PMB_sc_. The PBM_lcc_ curve shows a larger amplitude of change in inferred forest cover than the PFT and the PMB_sc_, linked to the fact that this method includes an ordinal stage of classification as well as numerical calculations. Reconstructed forest cover values for the PBM_lcc_ lie between the other two curves until the last millennium, when they decline rapidly.

The validity of these three curves may be assessed by comparing them to REVEALS reconstructions of forest cover for ~6000 yr BP, ~3000 yr BP and during the last 700 years^9^ (Fig. 2). Based on the PBM and PFT curves, the earliest of these time windows should correspond to the latter stages of the Europe-wide forest maximum. The REVEALS estimate of 73% forest cover for mid-latitude and northern Europe combined at ~6000 years ago lies between that for the PBM_sc_ (67%) and those for PFT (84%) and PBM_lcc_ (80%). At ~3000 yr BP, REVEALS indicates a decline in overall forest cover to 65%, in line with the three other methods of forest reconstruction. For the last 750 years, REVEALS shows forest diminishing further to between 46% and 51.5%, close to the values reconstructed using the PBM_sc_, but above that for PBM_lcc_ and below that for the PFT reconstruction. The REVEALS model also calculates standard errors (SEs) for estimates of plant cover. Because the mean REVEALS estimates used in this paper are calculated from a large number of grid-based mean forest cover estimates with their SEs for each time slice, we approximate the error on the REVEALS mean estimates used in this paper to be ca. ± 10% of reconstructed values (i.e. forest cover between 66% and 80% at 6000 yr BP). The PFT and PBM mostly lie within the SEs of the REVEALS estimates, but towards their maximum ends, e.g. PBM_sc_ close to −10%, and PBM_lcc_ and PFT close to +10%. The trends are comparable for northern Europe alone, but are different for mid-latitude Europe alone, with PFT values close to PBM_sc_ and REVEALS.

A second means of testing the different forest reconstructions is to examine how well they match modern forest cover for the same grid cells. While remotely sensed estimates of forest might be expected to offer the clearest results and the most rigorous test, in fact the Corine and Forest Map 2006 data have strongly different outcomes; i.e. 45% and 29% modern forest cover, respectively. This inconsistency partly reflects the ontological question of “what is a forest?” Corine uses distinct land-cover classes, and land classified as forest may include some open areas as the minimum required crown cover for a forested class is only 30%. The Forest Map 2006 is based on a minimum 50% tree crown cover with 5 m used as a minimum height of trees^28^. It also highlights the epistemological problem that differences in spatial resolution of measurement can fundamentally alter results^30^, in this case between 25 m and 100 m measured spatial resolution. An alternative data source for modern forest cover derives from surface pollen samples. We have transformed the surface pollen data set for Europe^27^ using both variants of the PBM, which leads to modern forest cover estimates of 49% (PBM_sc_) and 54% (PBM_lcc_). Overall, most estimates of modern forest cover for the grid cells used by Trondman *et al*.^10^ are between 45% and 49%; that is, close to that reconstructed for the 100 to −65 BP REVEALS time window.

**Figure 3:**
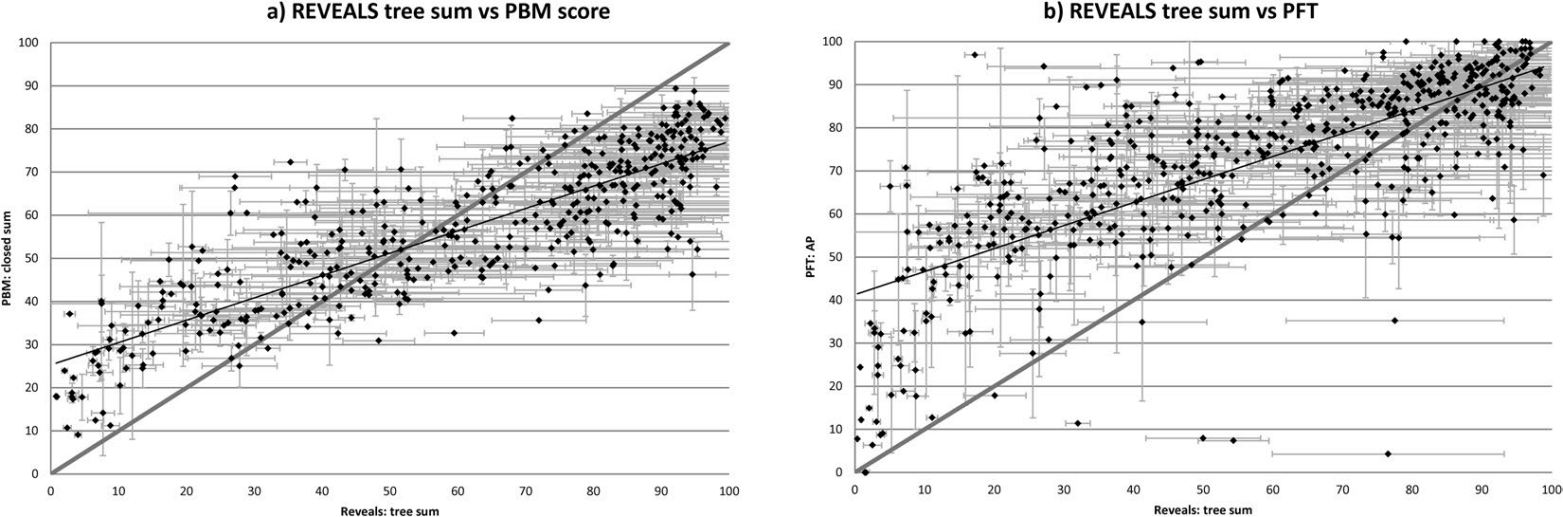
Major axis regression between forest cover estimates for different pollen-based methods by grid cell; (**a**) REVEALS tree sum vs PBM score; (**b**) REVEALS tree sum vs PFT.

A test of the different semi-quantitative reconstruction methods (i.e. PBM, PFT) is provided by comparison with the REVEALS forest estimates for the five different time windows. We use here standardized major axis regression as standard correlation methods and assume a dependent relationship between the variables under consideration. The statistical comparison shows an *r* value from standardized major axis regression of 0.69 between REVEALS and PBM_sc_ forest values (Fig. 3). The slope of the regression line for the PBM_sc_ is shallower than the 1 to 1 line, and the relationship may not be linear; PBM_sc_ consequently underestimates forest cover in densely wooded landscapes, but overestimates it in open landscapes, relative to REVEALS. This is typical of the relationship between vegetation and pollen percentage data, due to extra-regional background pollen influx^31–34^. This tendency can be seen in forest reconstruction for individual pollen records, eight of which are shown in Fig. SI-2. Comparison between REVEALS and PBM_sc_ reconstructions of forest cover for these records shows similar overall trends, but with REVEALS having a higher proportion of forest in densely-wooded landscapes (e.g. southern Scandinavia) and a lower proportion in open landscapes (e.g. Scotland). This appears to confirm the pattern shown in Fig. 3, namely that PBM_sc_ does not capture the true range of variation in landscape openness, and underestimates past forest extent for the period of the forest maximum.

For the PFT %AP the standardized major axis regression gives *r* = 0.47. There is a good match with REVEALS estimates at the top end of the range (i.e. for dense forest) but with increasing divergence between the two as landscapes become more open.

## Discussion

The overall long-term trend in forest cover across northern, central and western Europe was one of an early Holocene expansion to a forest maximum, followed by gradual decline during the second half of the Holocene. Pollen-based reconstructions indicate significant differences between mid-latitude and northern Europe in terms of the magnitude and timing of forest loss. The PFT method indicates that the forest maximum occurred earlier in northern than in mid-latitude Europe, at 8000 ± 500 yr BP compared to 6000 ± 1000 yr BP, although this is less clear in PBM_sc_ or PBM_lcc_. The decline in forest cover in northern Europe between 8000 and 6000 yr BP preceded the arrival of agriculture here. Consequently, it is not clear whether this trend is the result of cultural factors, such as Mesolithic burning^32^ or natural ones, such as the precessionally-induced increase in net incoming solar radiation during summer months at high latitudes during the early Holocene. The extra-tropical Northern Hemisphere thermal optimum (9700–7000 yr BP^33^) is well-attested in northern Scandinavia^35,36^, and broadly matches the forest maximum in Europe’s northern boreal zone (Fig. SI-3), but with some time lag. PFT and PBM_sc_ suggest that the boreal forest extent changed relatively little between ~5000 and 1700 yr BP, notwithstanding important shifts in its species composition during the second half of the Holocene^37^. After 1700 yr BP, boreal forest cover decreased relatively rapidly towards modern values of around 55–60%, consistent with archaeological and historical evidence of increasing human impact on the landscapes of northern Europe, for example associated with Norse expansion^38^.

The maximum spatially-averaged forest cover across mid-latitude Europe appears to have been slightly lower than it was in northern Europe, at around 70%, although this disguises important regional variations. The western Atlantic seaboard of France, Britain and Ireland, for example, was always much less densely forested than central Europe^12,19,21,39^. Mid-latitude forests began to decline in extent soon after ~6000 BP, by which time Neolithic farming was established across the whole of mid-latitude Europe^40^. In some regions, such as Britain, it has been possible to establish a clear linkage between early Neolithic settlement and initial forest conversion to agricultural land via pollen and archaeological data sets^41^. In other regions, such as southern Germany, early Neolithic settlement is associated with an increase in secondary woodland, but no diminution in overall forest cover^42^. Mid-latitude forests are dominated by broad-leaf, deciduous trees, typically developed on brown earth soils, along with needle-leaf trees in upland regions. In consequence, most mid-latitude lowland forests occupied land that was edaphically and climatically well suited for conversion to fields and pastures. Pollen-based reconstructions indicate that forest conversion to agricultural land becomes visible palynologically at a sub-continental scale during late Neolithic and Bronze Age times, during the later 5^th^ and 4^th^ millennia BP. The loss of forests was well under way by 3000 yr BP, with REVEALS estimates for mid-latitude Europe indicating a fall to ~63% from the mid-Holocene forest maximum. In other words, around one-fifth of the total net loss of temperate forest occurred prior to the late Bronze Age. Thus it is clear that anthropogenic impact on Europe’s temperate deciduous forests was already substantial prior to Iron Age times^10,19,21^ alongside that of climatic change^43^.

The decline in mid-latitude forest cover continued through Iron Age and Roman times, but was halted or reversed in the post-Roman Migration period, around 1700–1300 yr BP. The PBM reconstructions show stabilization and the PFT reconstruction a slight increase in forest cover during this period when there were mass movements of human populations across temperate Europe^44^. Pollen data therefore confirm that the post-Roman decline in rural settlement was accompanied by re-growth of secondary forests in many regions. After 1300 yr BP, the long-term decline in mid-latitude forest cover was renewed, and at an accelerating rate. This coincided with the “grand défrichements” of Mediaeval times, when forests were again cleared to make way for the *œcumene* of farms and villages, especially in north central Europe^45^, pp. 102 ff.). The transformation of forests into cultural landscapes during Mediaeval and later times is well recorded in textual, place-name and other historical evidence^46^, and this provides a valuable source for cross-comparison with pollen-based land-cover reconstructions (e.g.^47^. For example, Schlüter^48^ used a variety of historical evidence to map the area of forested land across north central Europe prior to Mediaeval clearances and also for 1900 CE. In practice, most of the forest loss occurred prior to 1500 CE according to Devèse (cited in^49^. The reconstruction made by Schlüter indicates a fall from perhaps as much as 72% forest cover to 34% between pre-Mediaeval and modern times (Table SI-1). While Schlüter used 900 CE as a starting date, our pollen-based land-cover reconstructions indicate that forest clearance in north central Europe had already begun by 850 ± 100 CE (i.e. during Carolingian times), and we therefore use 550–750 CE as a pre-Mediaeval baseline for comparison. According to the PBM_sc_, forest cover at this time was ~60% for the same area, and declined to 45% for the most recent time window (post-1750 CE). The REVEALS estimate for the most recent time period (post-1850 CE) is 41%.

**Figure 4:**
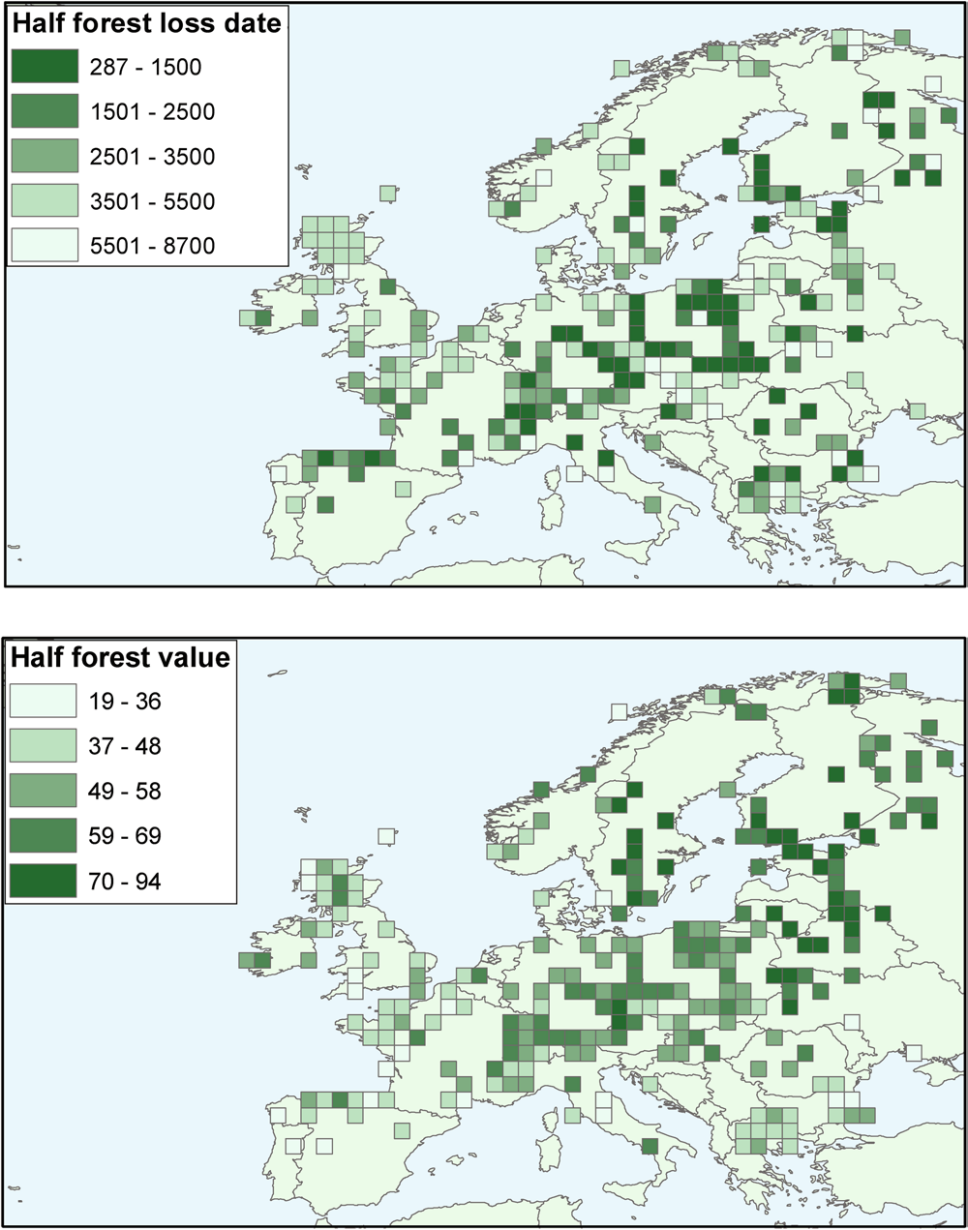
Mapped regional differences in timing of forest loss in calendar years BP. PBM_sc_ by grid cell for “half-life” (i.e. date of half-way point between forest cover maximum and minimum). [map generated using ArcMap (version: 10.4) http://desktop.arcgis.com/en/arcmap/].

In order to highlight regional differences in the timing of deforestation, we have calculated a forest loss index using the PBM_sc_ for individual grid cells across mid-latitude Europe. This index uses the first date by which half of the forest cover was lost in each pollen sequence. This index is not of great value in areas that experienced little or no deforestation, such as many northern boreal forests. On the other hand, the 50% forest loss value is similar whether using the PBM_sc_ or REVEALS method (Fig. 3a), so should represent an approximate measure of forest removal. When mapped (Fig. 4) the forest loss index confirms marked inter-regional differences in the timing of deforestation, in particular between north central Europe, where the majority of forests remained intact until Mediaeval times, and north western Europe where most forests had already been cleared in Bronze and Iron Age times. Some other areas, such as coastal regions of northern Europe (e.g. southern Scandinavia) also experienced substantial forest loss during later prehistory. The early date for forest clearance in England is in accord with the Domesday record, which showed only 15% of the surveyed lands as still wooded in 1086 CE^49,50^.

The different methods compared in this study show good agreement concerning the timing of land-cover change, but differences in the extent of past forest area and magnitude of forest loss. In particular, PBM and PFT reconstructions may under-estimate the true amplitude of forest cover change when compared with REVEALS reconstructions. At a continental scale, Europe’s forests reached their maximum extent between ~8000 and ~6000 years ago, and have been in retreat since the mid-Holocene in mid-latitude Europe. The rate of forest loss accelerated after ~4000 yr BP associated with forest conversion to farming and grazing land during Bronze and Iron Age times. In contrast to the mid-latitude belt of temperate mixed deciduous forests, the needle-leaf forests of the northern boreal zone have been impacted much less by human land-use conversion and most of those impacts have occurred in the last two millennia. This is seen in the late dates of maximum forest clearance found in central Sweden, Finland, Estonia and north east Russia, apart from the most northerly sites where the main forest decline was early and possibly climate-driven.

The transformation of Europe’s landscape from wildwood to farmland therefore extends back well into prehistoric times. It is clear that by the late Bronze Age, agricultural communities had already made significant inroads into transforming the natural vegetation and beginning a process that was accelerated through late prehistory and historic times. This has major significance for understanding long-term forest dynamics and for the future conservation and management of Europe’s common natural heritage.

## Methods

We used pollen-inferred forest-cover reconstructions for the Holocene described by Trondman *et al*.^10^, Marquer *et al*.^21^ and Fyfe *et al*.^19^ for REVEALS, Fyfe *et al*.^12^ for PBM, and Davis *et al*.^16^ for PFT. Figure SI-1 shows further details of the PBM and REVEALS methods. For this comparison we have used the most consistent REVEALS estimates of woodland cover available in terms of methodology used, i.e. the grid-based reconstructions of Trondman *et al*.^10^. These reconstructions are based on the combined pollen records from similar-sized areas, i.e. 1 × 1° grid-cells, while the existing time-continuous REVEALS reconstructions were performed using records from generally larger and different-sized areas^4,10,19,21^. This choice means that REVEALS estimates are available only for the five time windows selected in Trondman *et al*.^10^. In order to compare them statistically (Fig. 3), data were averaged for each of the 230 1 × 1 degree grid cells, used in^10^, and re-sampled on a common time interval. As well as this data set for inter-comparison of the different methods, our PBM and PFT reconstructions also include a second, larger data set that incorporates pollen sites and grid cells not covered by the REVEALS reconstructions. The data used for this inter-comparison derive from, and can be accessed via, the European Pollen Database (EPD)^23^, supplemented in the case of REVEALS and PFT by some additional data as described in^10,16^, respectively. All the pollen records used have calibrated ^14^C-based chronologies^12,23^ and meet the minimum dating standard set out by^51^.

For the REVEALS and PFT-based estimates, we use the sum of arboreal taxa as a proxy for forest cover. For the PBM approach, two different values are provided, which are related to different stages in application of the method (see Fig. SI-1a). These are firstly, the closed sum, as calculated by summing scores for needle-leaf and broad-leaf forest (= PBM score abbreviated PBM_sc_); secondly, % of samples assigned to each Land Cover Class, specifically the percentage assigned to needle-leaf or broad-leaf forest combined with half of the semi-open categories (= LCC assigned, abbreviated PBM_lcc_). The latter follows^14^, where an improved match between the pollen-based and remotely sensed data was found through re-assigning samples from ‘semi-open’ classes.

We have reconstructed forest changes in two regions, firstly, the mixed temperate hardwood forest zone of mid-latitude Europe, and secondly, the boreal needle-leaf forest zone of Northern Europe (Fig. 1). The changes described here are the net aggregate balance between forest gain and loss, not gross changes of total gain/loss. Consequently, substantially more “old growth” forest will have been lost than the net change in forest cover calculated between different time periods suggests.

The forest loss index uses the first date by which half of the forest cover had been lost in each pollen sequence, calculated as the mid-point between the maximum and minimum tree cover value (using the PBM_sc_), with records only being utilised if they span both the early and late Holocene.

### Data availability

The primary datasets analysed during the current study are available via the European Pollen Database repository, http://www.europeanpollendatabase.net/ and the European surface pollen database at http://www.europeanpollendatabase.net/wiki/doku.php?id=empd_download_database. The transformed results using the PBM are available in PANGAEA at https://doi.pangaea.de/10.1594/PANGAEA.853947. The REVEALS data are in the process of being archived in PANGAEA.

## Electronic supplementary material

Supplementary information
