# Supplementary material for: Europe’s lost forests: a pollen-based synthesis for the last 11,000 years
Source: Sci Rep. 2018 Jan 15;8:716. doi: 10.1038/s41598-017-18646-7 (PMC5768782; doi:10.1038/s41598-017-18646-7)
Supplement: Supplementary file 1 — Supplementary information [file 41598_2017_18646_MOESM1_ESM.doc]

Europe’s lost forests: a pollen-based synthesis for the last 11,000 years: Supplementary files

^1^Roberts, N., ^1^Fyfe, R.M., ^1^Woodbridge, J., ^2^Gaillard, M-J., ^3^Davis, B.A.S., ^3,8^Kaplan, J.O., ^4^Marquer, L., ^5^Mazier, F., ^4^Nielsen, A.B., ^6^Sugita, S., ^2^Trondman, A.-K. and ^7^Leydet, M.

^1^School of Geography, Earth and Environmental Sciences, Plymouth University, Drake Circus, Plymouth, UK

^2^School of Natural Sciences, Linnaeus University, Barlastgatan 11, Kalmar, Sweden

^3^ Max Planck Institute for the Science of Human History, Jena, Germany

^4^Department of Physical Geography and Ecosystem Science, Lund University, Sölvegatan, Lund, Sweden

^5^Bureau C229 Research House of Jean Jaurès University, 5, Allées A. Machado, Toulouse, France

^6^Institute of Ecology, Tallinn University, Narva mnt 25, Tallinn, Estonia

^7^Institut Méditerranéen de Biodiversité et d'Ecologie marine et continentale, Aix-Marseille Université, Marseille

^8^ARVE Research SARL, Pully, Switzerland

Corresponding author: Neil Roberts: C.N.Roberts@plymouth.ac.uk

Figure SI-1a: Flow chart to show PMB_sc_ vs % PBM_lcc_; 1b: Flow chart for the application of the REVEALS model (Sugita 2007) to the LANDCLIM dataset (Trondman et al 2015, Marquer et al 2014)


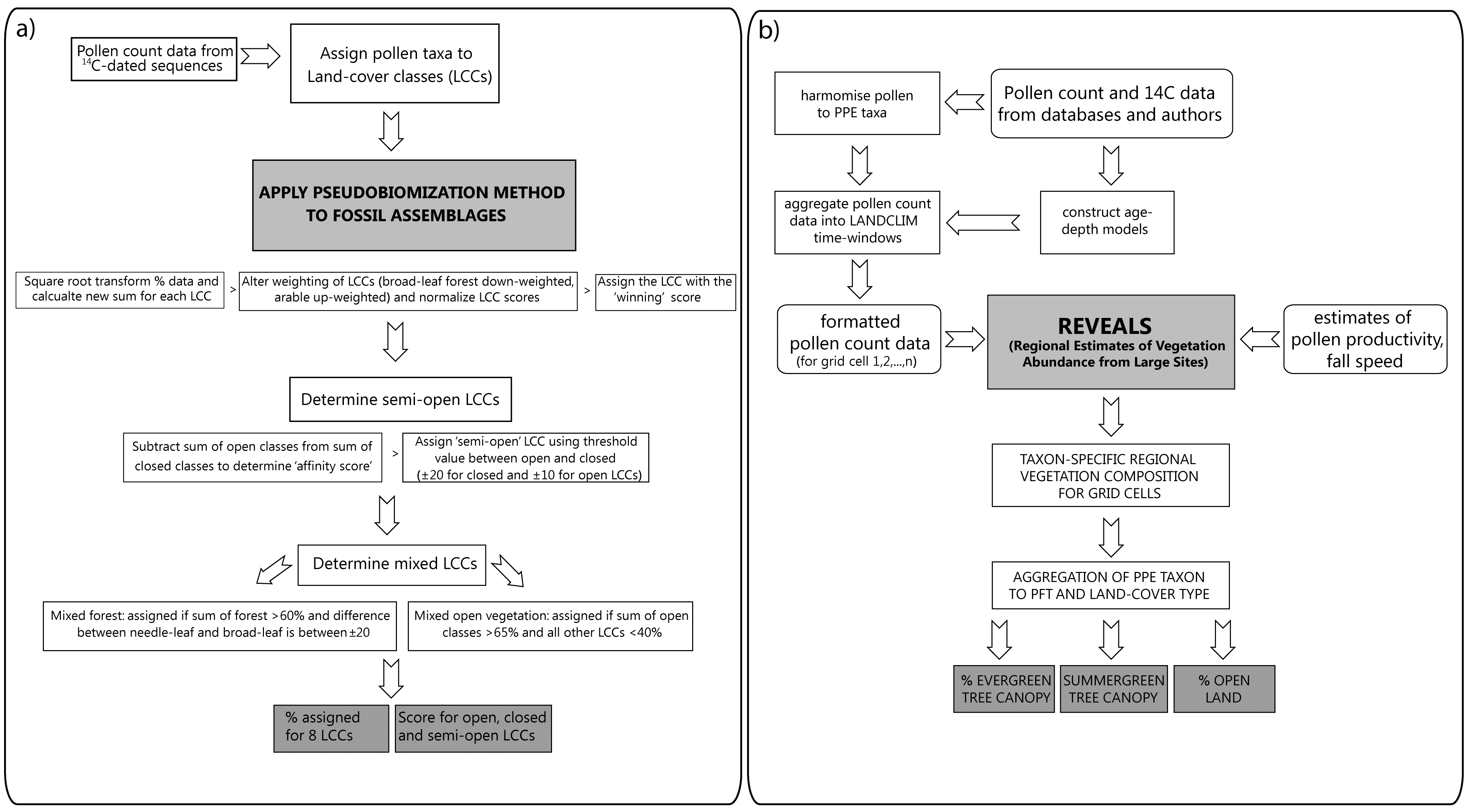


Figure SI-2: REVEALS vs PBM_sc_ for selected individual pollen records from Scotland and the circum-Baltic region (REVEALS data from Marquer et al 2014, Fyfe et al 2013). Note how the PBM_sc_ often underestimates forest cover in densely wooded landscapes, but overestimates it in open landscapes, relative to REVEALS.


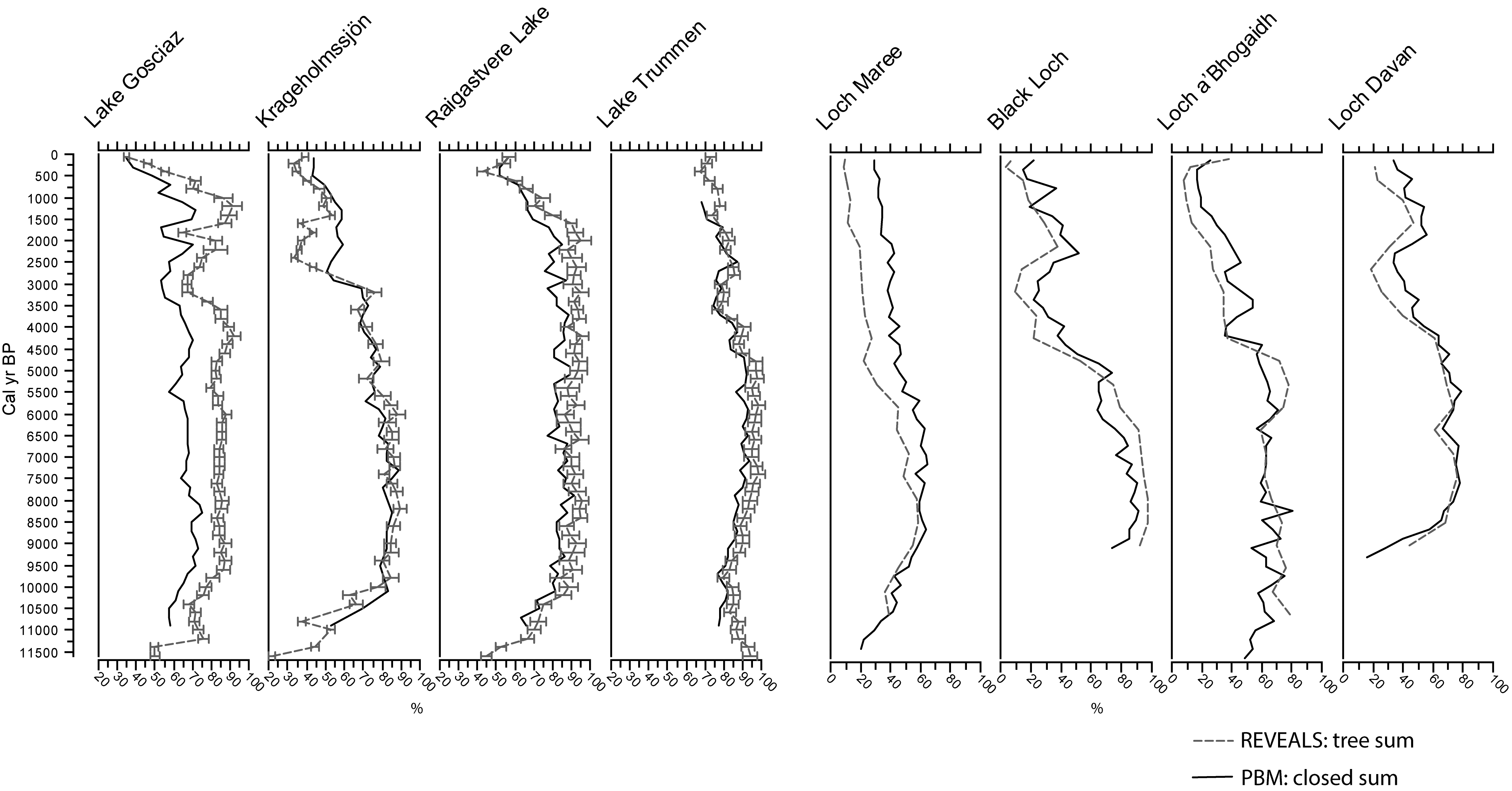


Figure SI-3: Pollen-inferred forest cover for mid-latitude and northern Europe during the last 11,000 years (REVEALS grid cells only, figure 2 for full key) compared to Northern Hemisphere extra-tropical temperature change stack of Marcott et al. (2013). Temperature data from http://science.sciencemag.org/content/suppl/2013/03/07/339.6124.1198.DC1


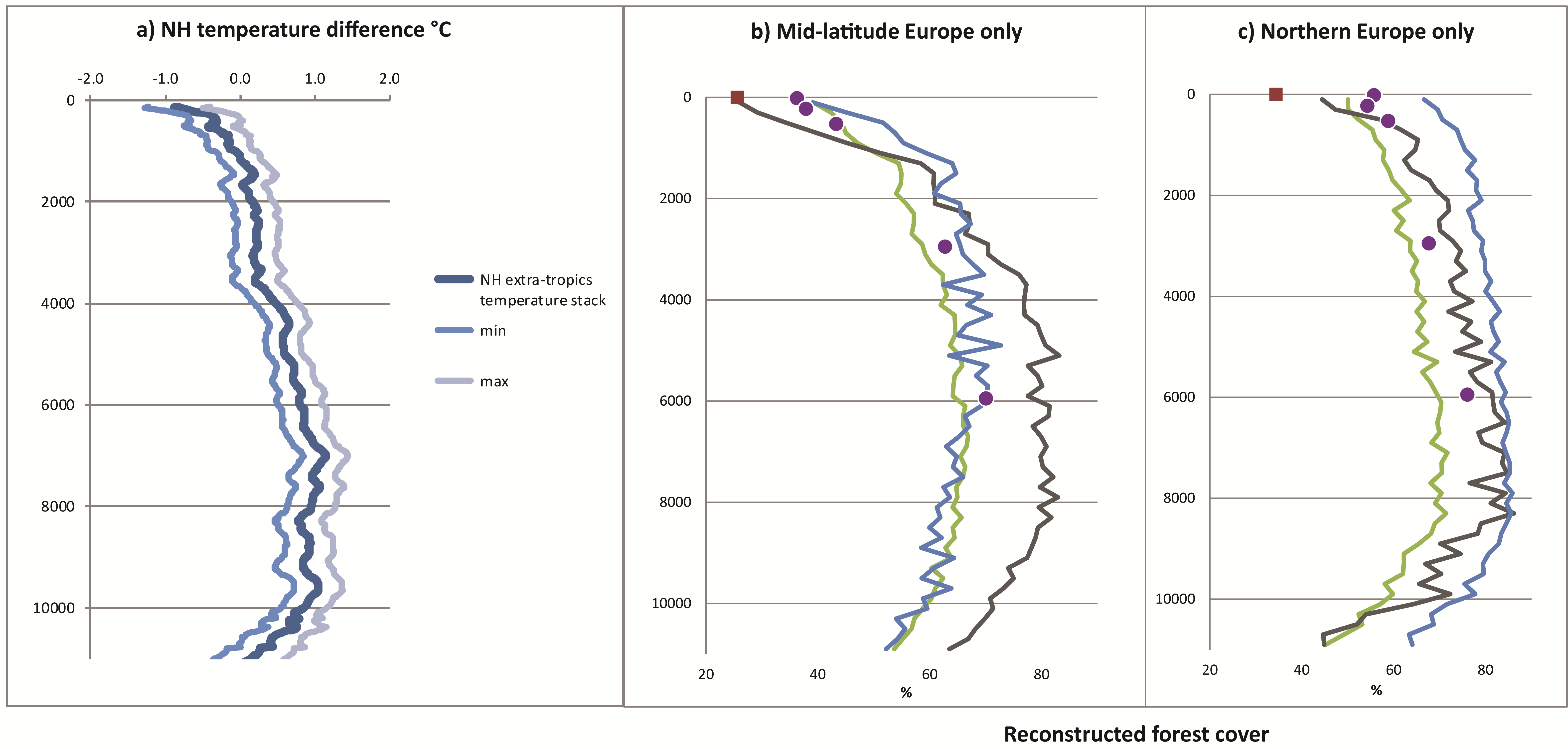


Table SI-1: Reconstructed forest cover for north central Europe in recent and early Mediaeval times using different data sources and methods. Forest cover maps from historical documentary sources from Schlüter (1952)

| **Time window** | **Historic maps** | **Time window** | **PFT** | **Time window** | **PBM_sc_** | **PBM_lcc_** | **Time window** | **REVEALS** |
| --- | --- | --- | --- | --- | --- | --- | --- | --- |
| *AD 1900 (50 BP)* | 33.65 % | *200- -60 BP* | 60.95 % | *200- -60 BP* | 44.79 % | 11.18 % | *100- -65 BP* | 40.82 % |
| *Early Mediaeval (1500-1000 BP)* | 71.89 % | *1500-1300 BP* | 79.76 % | *1500-1300 BP* | 60.28 % | 52.21 % | No data | |

**References**

Fyfe, R.M., Twiddle, C., Sugita, S., Gaillard, M.-J., Barratt, P., Caseldine, C.J., Dodson, J., Edwards, K., Farrell, M., Froyd, C., Grant, M.J., Huckerby, E., Innes, J.B., Shaw, H. & Waller, M. The Holocene vegetation cover of Britain and Ireland: overcoming problems of scale and discerning patterns of openness. *Quaternary Science Reviews*. **73**, 132-148 (2013).

Marcott, S.A., Shakun, J.D., Clark, P.U. & Mix, A.C. A reconstruction of regional and global temperature for the past 11,300 years. *Science*, *339*, 1198-1201 (2013).

Marquer, L., Gaillard, M.-J., Sugita, S. *et al.* Holocene changes in vegetation composition in northern Europe: why quantitative pollen-based vegetation reconstructions matter? *Quaternary Science Reviews*. **90**, 199–216 (2014).

Schlüter, O. *Atlas Ostliches Mittleuropa. Belefeld, Velhagen und Klasing* (plate 10) (1952)

Sugita, S. Theory of quantitative reconstruction of vegetation I: pollen from large lakes REVEALS regional vegetation composition. *The Holocene*. **17**, 229-241 (2007).

Trondman, A.-K., Gaillard, M.-J., Mazier, F.. *et al.* Pollen-based quantitative reconstructions of Holocene regional vegetation cover (plant-functional types and land-cover types) in Europe suitable for climate modelling. *Global Change Biology*. **21**, 676-697 (2015).
